# Warm Is Better: Incubation Temperature Influences Apparent Survival and Recruitment of Wood Ducks (*Aix sponsa*)

**DOI:** 10.1371/journal.pone.0047777

**Published:** 2012-10-15

**Authors:** Gary R. Hepp, Robert A. Kennamer

**Affiliations:** 1 School of Forestry and Wildlife Sciences, Auburn University, Auburn, Alabama, United States of America; 2 Savannah River Ecology Laboratory, Aiken, South Carolina, United States of America; University of Lausanne, Switzerland

## Abstract

Avian parents that physically incubate their eggs must balance demands of self-maintenance with providing the proper thermal environment for egg development. Low incubation temperatures can lengthen the incubation period and produce changes in neonate phenotype that may influence subsequent survival and reproduction. We artificially incubated wood duck (*Aix sponsa*) eggs at three temperature regimes (low, 35.0; mid, 35.9; and high, 37.3°C) that are within the range of temperatures of naturally-incubated nests. We tested the effect of incubation temperature on duckling body composition, fledging success, the probability that females were recruited to the breeding population, and their subsequent reproductive success. Incubation period was inversely related to incubation temperature, and body mass and lipid mass for newly-hatched ducklings incubated at the lowest temperature were lower than for ducklings produced at higher temperatures. In 2008, ducklings (*n* = 412) were individually marked and broods (*n* = 38) containing ducklings from each temperature treatment were placed with wild foster mothers within 24 hrs of hatching. Ducklings incubated at the lowest temperature were less likely to fledge from nest sites than ducklings incubated at the higher temperatures. We recaptured female ducklings as adults when they were either prospecting for nest sites (*n* = 171; 2009–2011) or nesting (*n* = 527; 2009–2012). Female ducklings incubated at the lowest temperature were less likely to survive and be recruited to the breeding population than females incubated at higher temperatures. Reproductive success of surviving females also was greater for females that had been incubated at warmer temperatures. To our knowledge, this is the first avian study to link developmental conditions experienced by neonates during incubation with their survival and recruitment to the breeding population, and subsequent reproductive success. These results advance our understanding of incubation as an important reproductive cost in birds and support the potential significance of incubation in influencing the evolution of avian life histories.

## Introduction

Incubating birds must balance the competing demands of self-maintenance with the thermal needs of developing embryos. Optimal growth and development of embryos takes place within a narrow range of incubation temperatures [1], [2], [3]. Reduced attendance by incubating parents can lower egg temperature, thereby slowing embryo development, and leading to longer incubation periods [4], [5]. Lengthy incubation periods can be costly because they result in greater exposure of nests to predators and influence offspring quality [6].

Early developmental conditions that influence offspring phenotype can play an important role in subsequent survival and reproduction [7]. Effects of incubation microclimate on hatchling phenotype and fitness characteristics have been well-studied in oviparous reptiles [8], [9], [10], but remain relatively unexplored in wild birds. However, this is changing. Low incubation temperatures in tree swallows (*Tachycineta bicolor*), for example, cause reductions in body mass and innate immunity of nestlings [11], [12]. Similarly, periodic cooling of eggs during incubation delays development and increases metabolic costs in zebra finches (*Taeniopygia guttata*) [13].

Wood ducks (*Aix sponsa*) nest in cavities, and females are strongly philopatric to natal areas, but males disperse [14]. Small reductions in incubation temperature lengthen the incubation period and influence phenotypes of newly-hatched wood ducks [3]. Recent experiments that artificially incubated wood duck eggs at three temperatures (35.0, 35.9, and 37.0°C), within the range of temperatures of naturally-incubated nests [3], showed ducklings incubated at 35°C differed in a variety of phenotypic characteristics. Ducklings incubated at the lowest temperature develop more slowly and expend more energy than ducklings incubated at higher temperatures [15]. After hatching, these ducklings also have reduced growth and acquired immune responses [16], higher baseline and stress-induced corticosterone levels [17], reduced locomotor performance [18], and expend more energy to thermoregulate [19] than ducklings incubated at higher temperatures (≥35.9°C). It is clear that the incubation environment of wood ducks and other birds can influence phenotypic quality in a number of important ways. Whether this phenotypic variation influences fitness of neonates currently is not known, but will have important implications for investment decisions made by incubating parents and the evolution of life histories (e.g., egg size and clutch size) [20].

In this study, we manipulated incubation period and neonate phenotype of wood ducks by artificially incubating eggs at temperatures similar to those used by Hepp et al. [3], DuRant et al. [16], [17], [18], [19], and Hopkins et al. [18]. Newly-hatched ducklings were individually marked, and broods containing ducklings from each of the incubation temperatures were placed in nests with foster mothers. We used recaptures of female ducklings as breeding adults to test the effect of incubation temperature on apparent survival and recruitment. We also monitored reproductive success of females over four breeding seasons. Because previous studies have shown incubation at 35°C negatively influenced a suite of phenotypic characteristics in young wood ducks, we predicted that females incubated at 35°C would be less likely to be recruited to the breeding population and have lower reproductive success than females incubated at higher temperatures.

## Materials and Methods

### Egg Collection and Incubation

**Figure 1:**
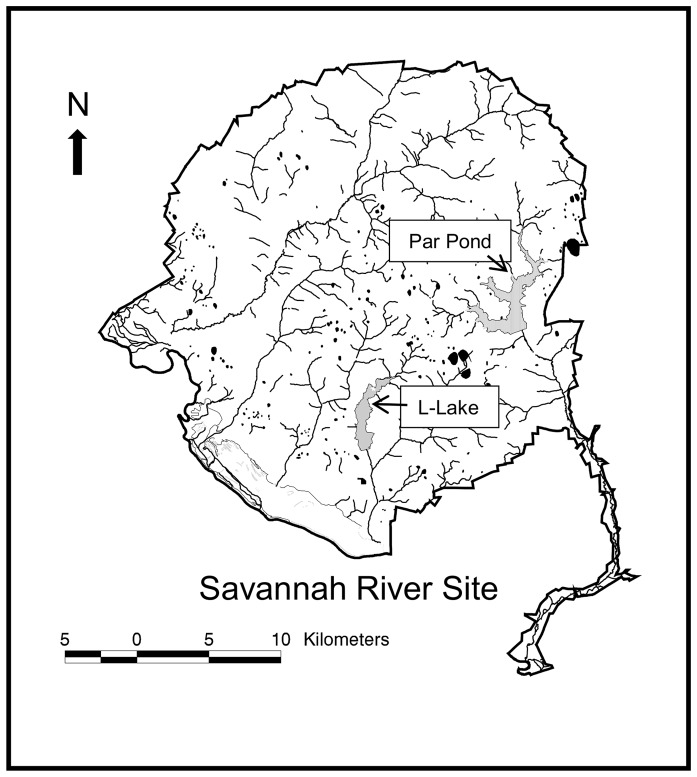
Department of Energy's Savannah River Site. The study was conducted at the Savannah River Site (800 km^2^) in west-central South Carolina. Wood ducks used nest boxes that were erected at Par Pond (*n* = 80; 1120 ha) and L-Lake (*n* = 30; 450 ha).

The study was conducted at the Department of Energy's Savannah River Site (SRS) in west-central South Carolina (33.1° N, 81.3° W; Figure 1). In 2008, we checked nest boxes on Par Pond (*n* = 80; 1120 ha) and L Lake (*n* = 30; 450 ha) every four days during the breeding season to locate new nests and designated three consecutive checks of all nest boxes as a ‘rotation’. Once located, active nests were visited daily, and fresh eggs were individually marked, removed, and replaced with wooden eggs to prevent nest abandonment. We weighed (0.01 g) fresh eggs on the day of collection, then stored eggs at 20°C and placed them in incubators (Grumbach model BSS 420, Lyon Technologies Inc.) at 4-day intervals. Eggs from nests initially discovered during the first, second, and third nest box inspection of a rotation were artificially incubated at low (35.0°C), mid (35.9°C), and high (37.3°C) temperature treatments, respectively. Incubators had two 1-hour cool down periods each day to simulate natural incubation behavior of wood ducks [21]. Incubator temperatures were recorded every 6 minutes using temperature data-loggers (HOBO® Pro V2, Onset Computer Corp.). We chose incubation temperatures similar to those of naturally incubated wood duck nests and that were known to produce differences in incubation periods of approximately 4 days between high-mid temperatures and mid-low temperatures [3]. This procedure allowed eggs collected during a rotation and incubated at different temperatures to hatch at approximately the same time.

### Marking Ducklings and Creating Broods

Incubated eggs were checked twice daily, and pipped eggs were moved to a single incubator to hatch (37.5°C and 80% humidity). Each pipped egg was placed in a PVC cylinder (10.6×10.6 cm) with mesh top to separate eggs and facilitate identification of ducklings after hatching. Incubation period (days), from placement into incubator until hatch, was recorded for each duckling. Newly-hatched ducklings were weighed (0.01 g) and tarsus was measured (0.01 mm) with digital calipers. Duckling sex was determined by cloacal examination and each individual was marked with a serially-numbered monel web tag [14]. Web-tagged ducklings were placed in holding boxes equipped with heat lamps. In 2008, we created broods comprised of ducklings from each of the three incubation temperature treatments. These broods were taken to nest boxes on Par Pond within 24-hr of hatching and placed with foster mothers that had been incubating wooden eggs.

### Duckling Body Composition

Randomly selected ducklings were removed from the hatching incubator for body composition analysis. Ducklings were sexed, weighed (0.01 g) and tarsus (0.01 mm) was measured with digital calipers. They were euthanized using CO_2_ inhalation and cervical dislocation, placed in individual bags, and frozen until processing. Ducklings were thawed and wet ducklings were dried (60–65°C) to constant mass. Dry ducklings were ground, and neutral lipids were extracted from whole ducklings in a Soxhlet apparatus using petroleum ether [22]. Lean samples were dried to constant mass, and lipid content was the difference in mass of the dry sample before and after extraction. Total lipid mass was calculated by multiplying the proportion of lipid in the dry, ground sample by dry mass of the duckling. Lean dry samples were weighed and burned in a muffle furnace at 550°C for 10 hr to determine ash content. Ash-free lean dry mass (AFLDM) was the difference between the ash content and lean dry samples, and the proportion of AFLDM in the sample was multiplied by dry mass of the duckling to obtain total AFLDM.

### Trapping, Recapture, and Reproductive Success

We used two trapping methods to recapture females that were originally web-tagged as ducklings. First, we equipped nest boxes on Par Pond and L Lake with nest box traps [23], and captured pre-nesting females that were prospecting for nest sites in mid-Jan and Feb 2009–2011. Female Wood Ducks prospect for nests in the morning; therefore, we set nest box traps in the afternoon and checked them the following day during late morning. Captured females were checked for web tags and leg bands, unmarked females were banded with USFWS leg bands, and all females were released at the nest site. We also checked nest boxes at Par Pond and L Lake for nesting activity every 4 days during the breeding seasons (Feb – July) of 2009–2012. All incubating females were captured at the nest during the first week of incubation and checked for web tags and leg bands. Unmarked females were banded with USFWS leg bands. We also followed the progress of each nest until it hatched or failed.

### Statistical Analysis

Data summaries and analyses were completed with SAS ver. 9.2 [24]. We used ANOVA to test for differences in incubation temperature among designated treatment groups. ANCOVA with fresh egg mass as the covariate was used to test the effects of incubation temperature treatment on incubation period, duckling mass, lipid mass, AFLDM, and tarsus length. Tukey tests were used following ANOVA and ANCOVA for separation of means and least squares means. We present means and least squares means ± SE.

Because fitness-related traits of ducklings like stress physiology, immune function, locomotor performance and growth rate [15], [16], [17], [18] and lipid mass (this study) do not differ for wood ducks incubated at temperatures ≥35.9°C, we pooled ducklings from high (37.3°C) and mid (35.9°C) temperature treatments to test the effect of incubation temperature on fledging and recruitment probabilities. We used Fisher's Exact Test to examine the effect of incubation temperature on fledging success and female return rates. Return rate was used as an index to female survival and recruitment and is biased low because probability of detection is <1. However, there is no reason to believe that detection probability should differ for female ducklings incubated at different temperatures. Further, we used the Live Recapture module in Program MARK [25] that uses the Cormack-Jolly-Seber (CJS) model to estimate capture probabilities of breeding female wood ducks on Par Pond and L Lake in 2007–2012. Capture probability is the probability that a marked female in our study population is captured. Estimated capture probability did not differ between years and was very high (0.96±0.02; Kennamer and Hepp unpublished data) indicating that trapping effort was consistent and most surviving females returned to nest boxes and were captured [26].

## Results

We hatched 412 ducklings (207 male and 205 female). Incubation temperature varied among treatment groups (*F*_2,408_ = 137,455, *p*<0.0001; Table 1). Fresh egg mass had no effect on incubation period (*F*_1,408_ = 0.07, *p* = 0.79), but warmer incubation temperatures shortened the incubation period of successive treatment groups by approximately 4 days (*F*_2,408_ = 1732.5, *p*<0.0001; Table 1). Body mass (*F*_1,407_ = 0.56, *p* = 0.45) and tarsus length (*F*_1,406_ = 2.97, *p* = 0.09) of newly-hatched ducklings did not differ by sex. Duckling body mass increased with fresh egg mass (*F*_1,407_ = 1827.6, *p*<0.0001), and ducklings that were incubated at low temperatures were lighter than ducklings incubated at mid and high temperatures (*F*_2,407_ = 11.09, *p*<0.0001; Table 1). Tarsus length also increased with fresh egg mass (*F*_1,406_ = 212.8, *p*<0.0001) and was greatest for ducklings incubated at low temperatures (*F*_2,406_ = 32.78, *p*<0.0001; Table 1).

**Table 1:** Means and least squares means (±SE) of incubation temperature (°C), incubation period (d), duckling mass (g), and tarsus length (mm) by temperature treatment of artificially-incubated wood duck eggs.

	Low	Mid	High
	*n* = 124	*n* = 139	*n* = 149
Incubation temperature	35.0 (0.002)c	35.9 (0.001)b	37.3 (0.005)a
Incubation perioda,b	37.9 (0.1)a	34.1 (0.1)b	30.1 (0.1)c
Duckling massa,b	26.08 (0.11)b	26.58 (0.11)a	26.78 (0.10)a
Tarsusa,b	18.82 (0.06)a	18.57 (0.06)b	18.16 (0.06)c

a Least-squares means are from ANCOVAs using fresh egg mass as the covariate.

b Means and least squares means within rows followed by different letters are significantly different (*p*<0.05).

**Figure 2:**
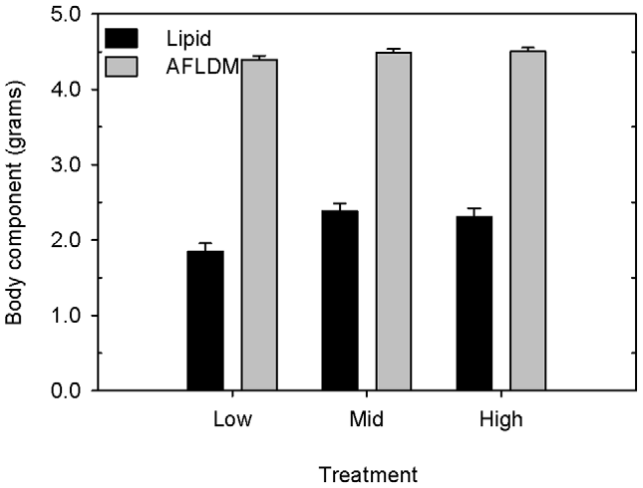
Body composition of ducklings. Least-squares mean (± SE) lipid mass and ash-free-lean-dry mass (AFLDM) of day-old wood duck ducklings that were artificially incubated at low (35.0°C; *n* = 15), mid (35.9°C; *n* = 16), and high (37.3°C; *n* = 14) temperatures. Fresh egg mass was used as the covariate in the ANCOVA. After adjusting for fresh egg mass, lipid mass of ducklings incubated at the low temperature was about 20% lzower than that of ducklings incubated at mid and high temperatures.

We examined body composition of newly-hatched ducklings (*n* = 45) that had been artificially incubated under similar conditions (low, mid, high temperatures; Figure 2). Ash-free lean dry mass (AFLDM) of ducklings increased with fresh egg mass (*F*_1,41_ = 279.17, *p*<0.0001), but fresh egg mass had little effect on duckling lipid mass (*F*_1,41_ = 1.63, *p* = 0.21). Incubation temperature affected lipid mass (*F*_2,41_ = 7.30, *p* = 0.002) but not AFLDM (*F*_2,41_ = 1.45, *p* = 0.25) of ducklings (Figure 2). Lipid mass of ducklings incubated at low temperatures (1.85±0.11 g) was approximately 20% lower than that of ducklings incubated at mid (2.38±0.10 g) and high (2.31±0.11 g) temperatures (Figure 2).

We created 38 broods from our ducklings (*n* = 412; Table 1). Every brood contained ducklings from each of the three incubation temperature treatments. Brood size averaged 10.8±0.2 ducklings, and the average delivery date of broods to foster mothers was 22 May±4 days. We checked nests for fledging success and found that 98% (404 of 412) of ducklings successfully left the nest (Table 2). However, ducklings incubated at higher temperatures were about 7 times more likely to fledge than ducklings incubated at the low temperature treatment (Fisher's Exact Test: right-sided *p* = 0.01; Odds Ratio  = 7.3; 95% CI  = 1.4–36.5; Table 2).

**Table 2:** Frequency that male and female wood duck ducklings fledged and left the nest after being incubated at high (≥35.9°C) and low (35.0°C) temperatures.

	Fledged		
	No	Yes	Total
Low temperature	6	118	124
High temperature^a^	2	286	288

a Includes ducklings from the mid and high temperature groups.

**Figure 3:**
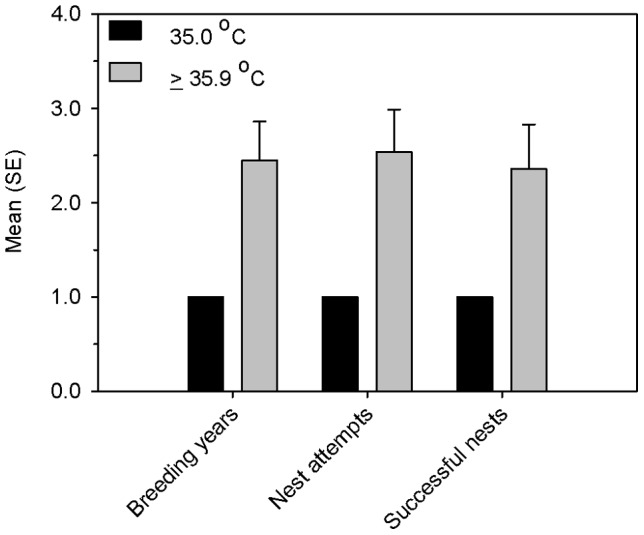
Relationship of incubation temperature to future reproductive success. Mean (± SE) numbers of breeding years, nest attempts and successful nests of female wood ducks that had been artificially incubated in 2008 at temperatures of 35°C (*n* = 1) and ≥35.9°C (*n* = 11). Nesting activity was monitored every four days in nest boxes (*n* = 110) at Par Pond and L Lake for four breeding seasons (2009–2012).

We used female ducklings that successfully fledged (*n* = 200) to test the effect of incubation temperature on apparent survival and recruitment of females to the breeding population. In 2009–2011, we captured pre-nesting female Wood Ducks (*n* = 171 individuals) in nest boxes when they were prospecting for nest sites. We also monitored nest boxes every 4 days during the breeding seasons of 2009–2012 and captured incubating females on the nest (*n* = 527 individuals). Using these methods, we recaptured 12 females that were marked as ducklings and placed with foster mothers. Recaptures of experimental ducklings occurred only in nest boxes on Par Pond; none were recaptured on L Lake. Most (10 of 12) females returned to nest within the same section of Par Pond as their foster mother's nest box. Five females were first recaptured prospecting for nest sites, but all females were eventually captured incubating nests. Seven females were first captured nesting in 2009, four females were captured nesting in 2010, and one female first nested in 2011. Small sample size reduced the power of our inference, but female ducklings incubated at the higher temperatures were about 4 times more likely to be recaptured as breeding adults as ducklings incubated at the low temperature (Fisher's Exact Test: right-sided *p* = 0.12; Odds Ratio  = 4.3, 95% CI  = 0.5–34.3; Table 3). We monitored nesting activity for four breeding seasons (2009–2012), and females incubated at higher temperatures had greater reproductive success (Figure 3).

## Discussion

Our study shows that small differences in incubation temperature can influence neonate phenotype and the probabilities that wood duck ducklings will fledge and be recruited to the breeding population. Foster broods were comprised of ducklings from each of the incubation temperatures and allowed effects of parental quality to be separated from that of incubation temperature. Small sample size reduced the power of our inferences, but we estimated that ducklings incubated at temperatures ≥35.9°C were 7 times more likely to fledge and 4 times more likely to be recruited as ducklings incubated at 35°C. The return rate of females (6.0%; 12 of 200) was low but was similar to that of wood duck females naturally-incubated and web-tagged previously at nest sites on the SRS; range: 2.2–6.8% [14]. Further, we monitored these females for four breeding seasons, and females that had been incubated at temperatures ≥35.9°C initiated more nests and completed more successful nests than those incubated at 35°C. We show that thermal conditions experienced by embryos during early development have important effects on subsequent survival and reproduction [7], [27].

**Table 3:** Frequency that female wood duck ducklings survived and were recaptured as breeding adults after being incubated at high (≥35.9°C) and low (35.0°C) temperatures.

	Survived		
	No	Yes	Total
Low temperature	53	1	54
High temperature^a^	135	11	146

a Includes ducklings from the mid and high temperature groups.

It is becoming clear that incubation is an important reproductive cost in birds [6], [28]. Incubating parents need to balance the competing demands of maintaining their body condition with the thermal requirements of developing embryos. This trade-off is more difficult to balance for species, like wood ducks, in which only one parent incubates. Studies that manipulate energy demands of incubating parents have provided strong evidence that incubation represents an important energetic constraint in species like European starlings (*Sturnus vulgaris*) and tree swallows [11], [29], [30]. When incubating parents are energetically constrained, incubation temperature can fall below levels needed for optimal embryo development [31]. Female wood ducks, for example, show restraint during incubation and favor self-maintenance over maintaining optimal thermal conditions for embryos [32].

In this study, low incubation temperature extends the incubation period of wood ducks and produces ducklings with reduced body mass and lipid mass. Reduced incubation temperature also lengthens the incubation period in a variety of other species of birds and influences traits potentially important for offspring survival [5], [12], [13]. Eggs incubated at low temperatures, for example, use more energy during development [33], [34] and young hatch with smaller nutrient reserves [35]. Similarly, DuRant et al. [15] reported wood ducks incubated at 35°C expended 20–37% more energy during development than did embryos incubated at higher temperatures. This increased energy expenditure by developing embryos is consistent with the 20% reduction in lipid mass we found in this study for newly-hatched ducklings incubated at 35°C. Nutrients that remain after hatching help to satisfy early energy requirements and are especially important to precocial young. Large residual nutrient reserves are helpful, for example, when food is unavailable or when young have to travel from nesting sites to distant brood-rearing areas. Body mass of newly-hatched ducklings, for example, frequently has a positive effect on survival [36], [37], [38].

Reduced residual energy reserves also may impact future growth and development of precocial young. For example, young wood ducks incubated at higher temperatures grew faster and were in better condition 9 days after hatching than ducklings incubated at low temperatures (35°C) [17]. Further, even though wood duck ducklings incubated at low temperatures have less residual energy, DuRant et al. [19] found they need to expend more energy to maintain their body temperature during thermal challenges than individuals incubated at higher temperatures. The ability to thermoregulate is especially important for young birds, and hypothermia is often an important source of mortality for young ducklings [39]. Indeed, many studies show that mortality of young ducklings, including wood ducks, is greatest within the first two weeks of hatching [40]. It is during this time that precocial young are transitioning to homeothermy, and when they may be most susceptible to hypothermia. Therefore, the negative relationship between low incubation temperature and duckling survival may partly reflect the effect that reduced residual nutrients have on an individual's ability to develop homeothermy and meet thermoregulatory challenges [19].

The ability to mount a strong immune response also can influence survival in young birds [41]. Early developmental conditions, like incubation, can influence phenotypes and potentially affect immune response in birds. Recently, cool incubation temperatures have been shown to lower innate immunity in tree swallows [12]. Further, DuRant et al. [16] found that the immune responses of young wood ducks incubated at low incubation temperatures were weaker than duckling incubated at higher temperatures. Reduced immune responses of young wood ducks likely make them more susceptible to diseases and parasites, potentially affecting their survival.

Incubation microclimate can produce significant changes to avian phenotypes; however, the relationship between these changes and components of offspring fitness is poorly understood. This is the first avian study that links actual incubation conditions to fitness consequences. Incubation temperature influences duckling phenotype in multiple ways; however, the mechanism by which differences in temperature affect survival and reproduction is unclear and requires further study. Trade-offs in energetic allocation by incubating parents can influence fitness of parents as well as offspring. As such, incubation is an important reproductive cost that likely has played an important role in the development of life history characteristics of birds like clutch size and egg size [20], [42].
